# *Rhinacanthus nasutus* Extracts Prevent Glutamate and Amyloid-β Neurotoxicity in HT-22 Mouse Hippocampal Cells: Possible Active Compounds Include Lupeol, Stigmasterol and β-Sitosterol

**DOI:** 10.3390/ijms13045074

**Published:** 2012-04-23

**Authors:** James M. Brimson, Sirikalaya J. Brimson, Christopher A. Brimson, Varaporn Rakkhitawatthana, Tewin Tencomnao

**Affiliations:** 1 Department of Clinical Chemistry, Faculty of Allied Health Sciences, Chulalongkorn University, 154 Rama I Rd, Pathumwan, Bangkok 10330, Thailand; E-Mails: james.b@chula.ac.th (J.M.B.); nroparav@gmail.com (V.R.); 2 Department of Clinical Microscopy, Faculty of Allied Health Sciences, Chulalongkorn University, 154 Rama I Rd, Pathumwan, Bangkok 10330, Thailand; E-Mail: sirikalaya.j@chula.ac.th; 3 Department of Biology and Biochemistry, Faculty of Science, University of Bath, Bath BA2 7AY, UK; E-Mail: Cab40@bath.ac.uk

**Keywords:** Alzheimer’s disease, glutamate, amyloid-β, neuron degeneration, oxidative stress, herbal medicine

## Abstract

The Herb *Rhinacanthus nasutus* (L.) Kurz, which is native to Thailand and Southeast Asia, has become known for its antioxidant properties. Neuronal loss in a number of diseases including Alzheimer’s disease is thought to result, in part, from oxidative stress. Glutamate causes cell death in the mouse hippocampal cell line, HT-22, by unbalancing redox homeostasis, brought about by a reduction in glutathione levels, and amyloid-β has been shown to induce reactive oxygen species (ROS) production. Here in, we show that ethanol extracts of *R. nasutus* leaf and root are capable of dose dependently attenuating the neuron cell death caused by both glutamate and amyloid-β treatment. We used free radical scavenging assays to measure the extracts antioxidant activities and as well as quantifying phenolic, flavonoid and sterol content. Molecules found in *R. nasutus*, lupeol, stigmasterol and β-sitosterol are protective against glutamate toxicity.

## 1. Introduction

*Rhinacanthus nasutus* (L.) Kurz (Acanthaceae) is an herb native to Thailand and Southeast Asia and is known for its antioxidant properties. It is commonly known as Snake Jasmine due to the shape of its flowers and because the root has traditionally been used as an antidote against snake venom. The medicinal plant, which can be drunk as a tea or made into a balm and is also traditionally used to treat a range of disorders including ringworm and inflammatory disorders. Previous work from this laboratory has shown *R. nasutus* to protect skin cells against IFN-γ and TNF-α induced apoptosis, potentially through an antioxidant mechanism [1]. *R. nasutus* root extract has also been shown to have a hepatoprotective effect in rats treated with Aflatoxin B1, which causes its hepatotoxic effects by causing oxidative stress in liver cells, causing damage to DNA, proteins and lipids [2]. We have also previously shown that HT-22 cells are protected from the detrimental effects of hypoxia and reoxygenation by the ethanol extract of *R. nasutus* root [3] and we suspect that *R. nasutus* root extract provides this protection through an antioxidant mechanism.

Accumulation of reactive oxygen species (ROS) in the cell, resulting from an imbalance of enzymatic and non-enzymatic oxygen free radical production or destruction, leads to lipid peroxidation, protein oxidation, and DNA damage, which in turn can lead to cell death [4–8]. The brain is particularly vulnerable to oxidative stress due to its high metabolic turn over, high lipid content and relatively low levels of antioxidants [9,10]. Glutamate is an excitatory amino acid which plays a role in learning, memory and brain aging [11], and it can also cause excitotoxicity in neuron cells [12]. However, this potential for neurotoxicity is masked by mechanisms to uptake glutamate from the synaptic cleft [13]. Glutamate toxicity may be caused by one of two pathways, the classical pathway involves specific glutamate receptors, which are blocked by glutamate receptor antagonists [4]. The other pathway is independent of specific glutamate receptors, and involves the induction of an imbalance in antioxidant enzyme systems such as a reduction in glutathione levels. The depletion of intracellular glutathione is brought about by the competition of glutamate with cystine for a glutamate cystineantiporter, leading to an imbalance in the homeostasis of cystine, which is the precursor of glutathione [14]. There is increasing evidence that there is a neurotoxicity element to Alzheimer’s disease, and therefore providing protection against neurotoxicity may delay or slow the onset of the disease, as it has been suggested that if the onset could be delayed by five years there would be a 50% reduction in the number of Alzheimer’s disease cases [15]. Neuronal cell loss in Alzheimer’s disease is preceded by the buildup of amyloid-β plaques [16–18] and a number of studies have shown that amyloid-β treatment results in an increase in the production of ROS [19].

In this article we set out to investigate the protective effect *R. nasutus* root ethanol extract against glutamate and amyloid-β_25–35_ toxicity in cultured mouse clonal hippocampal (HT-22) cells. We also have investigated various other extracts from *R. nasutus* leaves, and a hexane extract of both leaf and root. We have identified *R. nasutus* as neuroprotective, against both glutamate and amyloid-β toxicity, furthermore we show that the triterpenoid, lupeol, and sterols, stigmasterol, β-sitoterol are found in *R. nasutus* and as well as the triterpenoid β-amyrin (which has previously been shown to have antidepressant properties [20,21]) cause protection against glutamate toxicity.

## 2. Results and Discussion

### 2.1. Rhinacanthus Nasutus and Its Antioxidant Properties

Having previously identified the ethanol extract of *R. nasutus* root as protective against cell death in a hypoxia model [3], we investigated some of the properties of various extracts of *R. nasutus.* Table 1 shows the radical scavenging capabilities of *R. nasutus* extracts from leaf and root extracted in either ethanol or hexane. The ethanol extract of *R. nasutus* leaf appears to have the most radical scavenging activity in all of the radical scavenging assays, whereas, the hexane extract of the *R. nasutus* leaf has the least scavenging activity in each of the assays preformed. The total phenolic content of the extracts was also measured using the Folin-Ciocalteu method (Table 2) with the extracts showing a similar pattern. The ethanol extract of the leaf contained the most total phenols (6.5 ± 0.12 GAE mg·g^−1^ extract) with the ethanol extract of the root having the second highest amount of total phenols (2.17 ± 0.15 GAE mg·g^−1^ extract) and the hexane extracts having the least (root 0.36 ± 0.11 GAE mg·g^−1^ extract and leaf 0.4 ± 0.01 GAE mg·g^−1^ extract). The flavonoid content of the extracts was measured using the aluminum chloride colorimetric method and rutin as a standard; the results are represented as milligrams of rutin equivalents (RE) per gram of dry extract. The ethanol extract of leaf had the greatest amount of flavonoids (1.15 ± 0.14 mg·g^−1^ RE) followed by the ethanol extract of the root (1.13 ± 0.17 mg·g^−1^ RE) and the hexane extracts had no detectable flavonoids. It is notable that the root extract has a greater proportion of flavonoids (approximately 52%) making up its total phenolic content compared to the ethanol extract of leaf (approximately 17%).

### 2.2. Protective Effects of *R. nasutus* Against Glutamate Toxicity in Mouse Hippocampus (HT-22) Cells

**Figure 1:**
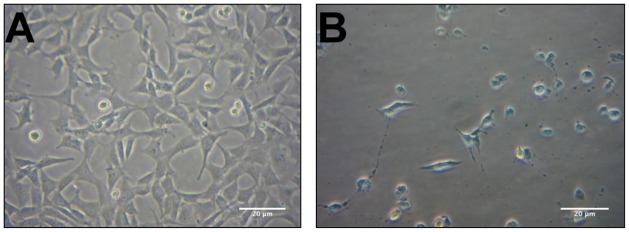

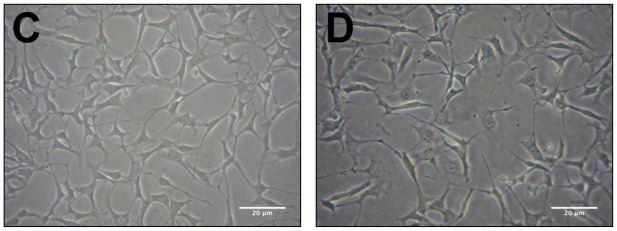
The protective effect of *R*hinacanthus *nasutus* ethanol extract against 5 mM glutamate. Phase contrast micrographs of HT-22 cells. (**A**) Control cells; (**B**) Glutamate 5 mM; (**C**) Glutamate 5 mM + *R. nasutus* root ethanol extract 10 μg; (**D**) *R. nasutus* root ethanol extract 10 μg.

HT-22 cells are a sub-clone of the immortalized HT4 cell line and are derived from mouse hippocampal cells. HT-22 cells are sensitive to glutamate, however, glutamate antagonists do not protect these cells from glutamate toxicity [22]. Along with the observation that antioxidants such as vitamin E are able to protect HT-22 cells against glutamate toxicity, these data imply that the cell death is occurring through an oxidative pathway [23]. The cell death caused by glutamate seen in HT-22 cells is a combination of both apoptosis and necrosis, with the necrosis occurring at relatively early time points (8–12 h) and apoptosis at later time points (16–24 h) [22]. Figure 1 shows phase contrast micrographs illustrating the detrimental effects of 5 mM glutamate on the HT-22 cell line after 24 h incubation (Figure 1B) compared to the control (Figure 1A). The glutamate treated cells have lost their shape and have started to detach from the surface. However, when the cells are treated with 10 μg·mL^−1^ ethanol extract of *R. nasutus* root, the cells maintain their structure and appear unharmed by the 5 mM glutamate (Figure 1C). Also 10 μg·mL^−1^ ethanol extract of *R. nasutus* root appears to have no detrimental effect on cells not treated with 5 mM glutamate (Figure 1D).

**Figure 2:**
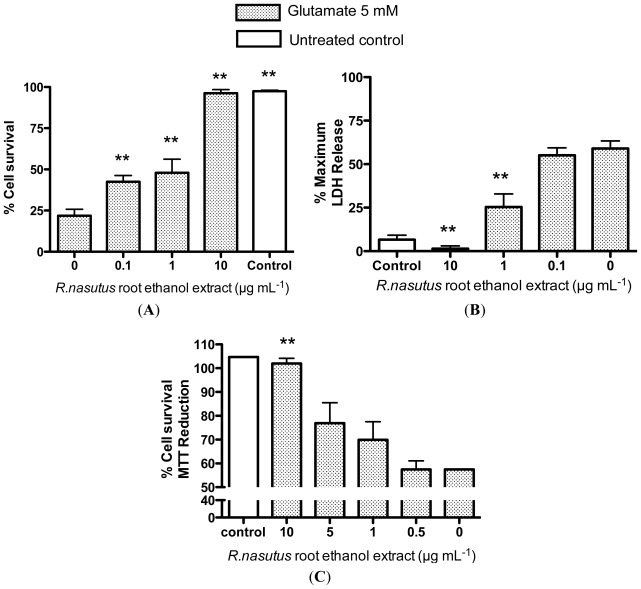
Quantification of HT-22 cell survival. (**A**) Trypan blue exclusion assay; (**B**) Lactate dehydrogenase release assay; (**C**) MTT assay. ** ANOVA dunnett’s *post hoc* test *p* < 0.01.

In order to measure the extent of the protection against glutamate toxicity that *R. nasutus* provides the HT-22 cells, the trypan blue exclusion assay (Figure 2A), the lactate dehydrogenase (LDH) assay (Figure 2B), and the MTT assay (Figure 2C) were employed. The trypan blue assay showed that cells treated overnight with glutamate (5 mM) resulted in an approximate 75% reduction in cell viability. When the cells were co-treated with 0.1–10 μg·mL^−1^ *R. nasutus* root ethanol extract, there was a significant increase in cell viability (ANOVA *post hoc p* value <0.01). The protective EC_50_ for *R. nasutus* root ethanol extract against glutamate (5 mM) in the trypan blue exclusion assay was 1.7 μg·mL^−1^ (95% CI 0.9 μg·mL^−1^ to 3.3 μg·mL^−1^). The LDH assay measures the release of lactate dehydrogenase from the cell through damage to the cell membrane. Treatment with glutamate (5 mM) overnight resulted in a 50% increase in LDH above control levels. Whereas co-treatment with *R. nasutus* root ethanol extract (1 and 10 μg·mL^−1^) resulted in a significant decrease in LDH release compared to glutamate alone (ANOVA *post hoc p* value <0.01). The protective EC_50_ in the LDH assay for *R. nasutus* is 0.63 μg·mL^−1^ (95% CI 0.35 to 1.1 μg·mL^−1^). In the MTT assay 5 mM treatment of the HT-22 cells resulted in a 43% reduction in cell viability and when co-treated with 10 μg·mL^−1^ *R. nasutus* root ethanol extract the viability was returned to control levels (ANOVA *post hoc p* value < 0.01). The protective EC_50_ in the MTT assay was 4 μg·mL^−1^ (95% CI 1.3 to 12 μg·mL^−1^).

**Figure 3:**
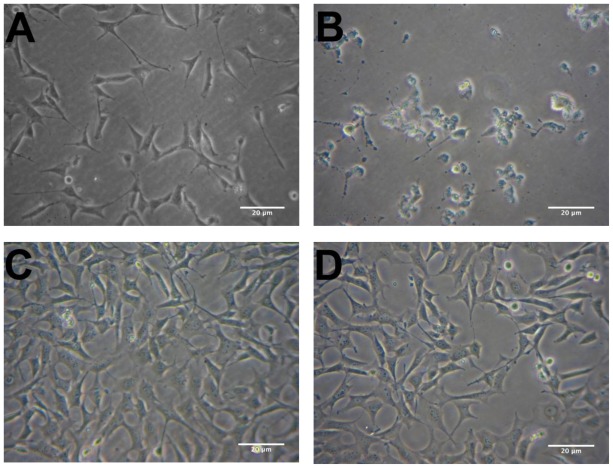
The protective effect of Hexane extract of *R. nasutus* root against 5 mM glutamate. Phase contrast micrographs of HT-22 cells. (**A**) Control cells; (**B**) Glutamate 5 mM; (**C**) Glutamate 5 mM + *R. nasutus* root hexane extract 10 μg·mL^−1^; (**D**) *R. nasutus* root hexane extract 10 μg·mL^−1^.

**Figure 4:**
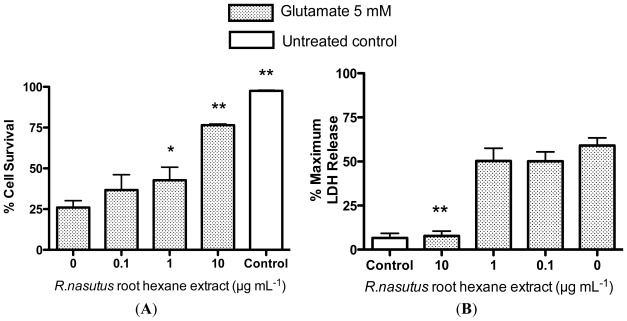
Quantification of HT-22 cell survival. (**A**) Trypan blue exclusion assay; (**B**) Lactate dehydrogenase release assay. * ANOVA Dunnett’s *post hoc* test *p* < 0.05; ** ANOVA Dunnett’s *post hoc* test *p* < 0.01.

Extraction using hexane, which is much less polar than ethanol, will dissolve more lipophilic compounds found in *R. nasutus*. Therefore, the hexane extract of *R. nasutus* was tested to see if it was more potent at providing protection for HT-22 cells against glutamate toxicity. HT-22 cells were treated with *R. nasutus* hexane extract and/or glutamate (5 mM) overnight or left untreated as a control. Figure 3A–D shows the protective effects of *R. nasutus* root hexane extract, viewed under 20 times magnification after overnight incubation with the stated treatments. The micrographs show the hexane extract of *R. nasutus* preventing the change in morphology and reduction in adherence to the surface that glutamate (5 mM) causes. The viability of the HT-22 cells was measured using the trypan blue exclusion assay (Figure 4A). Treatment with 5 mM glutamate resulted in a 75% reduction in cell viability (ANOVA *post hoc p* value < 0.01) compared to control cells. Co-treatment of HT-22 cells with 1 and 10 μg·mL^−1^ *R. nasutus* resulted in a significant increase in cell survival compared to glutamate alone (ANOVA *post hoc p* values < 0.05 and < 0.01 respectively). The resulting EC_50_ for the protection of HT-22 cells with the hexane extract of *R. nasutus* was 54.3 μg mL^−1^ (95% CI 10.9–269.8 μg mL^−1^). The extent of cellular damage and protection with *R. nasutus* hexane extract was further quantified using the LDH assay (Figure 4B). Treatment with 10 μg mL^−1^ *R. nasutus* resulted in a significant reduction of LDH release from the HT-22 cells after treatment with 5 mM glutamate (ANOVA *post hoc p* value <0.01).

**Figure 5:**
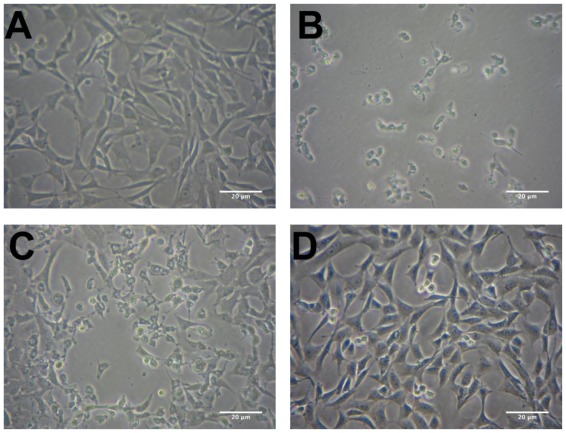
Protective effect of *R. nasutus* leaf ethanol extract against 5 mM glutamate. Phase contrast micrographs of HT-22 cells. (**A**) Control cells; (**B**) Glutamate 5 mM; (**C**) Glutamate 5 mM + *R. nasutus* leaf ethanol extract 10 μg·mL^−1^; (**D**) *R. nasutus* leaf ethanol extract 10 μg·mL^−1^.

**Figure 6:**
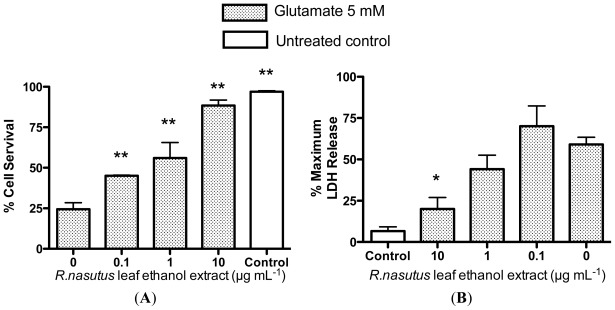
Quantification of HT-22 cell survival. (**A**) Trypan blue exclusion assay; (**B**) Lactate dehydrogenase release assay. * ANOVA Dunnett’s *post hoc* test *p* < 0.05; ** ANOVA Dunnett’s *post hoc* test *p* < 0.01.

Various studies have shown *R. nasutus* leaf extracts to have biological activities, including antioxidant activity [2,24,25], and since the data from the free radical scavenging assays suggest that the ethanol extract of *R. nasutus* leaf has the most potent radical scavenging capabilities (Table 1). We therefore tested the leaf ethanol extract for protective effects in HT-22 cells against glutamate toxicity. HT-22 cells were treated with *R. nasutus* leaf ethanol extract and/or glutamate (5 mM) overnight as well as a set of cells being left untreated. Figure 5C shows the protective effects of 10 μg mL^−1^ *R. nasutus* against the toxic effects of 5 mM glutamate in HT-22 cell. There appears to be some protection compared to glutamate alone (Figure 5B) although the cells do not appear like those in the untreated control (Figure 5A). *R. nasutus* leaf extract appears to have no toxic effect when added to HT-22 cells alone (Figure 5D). The cell viability of HT-22 cells was measured using the trypan blue exclusion assay (Figure 6A); Glutamate (5 mM) resulted in a significant 75% reduction in cell viability (ANOVA *post hoc p* value < 0.01). Whereas, co-treatment 0.1, 1 and 10 μg·mL^−1^ resulted in significant increases in cell viability (ANOVA *post hoc p* value < 0.01). The EC_50_ for the protection of cell viability by *R. nasutus* against 5 mM glutamate was 0.8 μg·mL^−1^ (95% CI 0.28–2.3 μg·mL^−1^). The extent of the cellular damage caused by glutamate (5 mM) in HT-22 cells was measured using the LDH assay (Figure 6B). Treatment of HT-22 cells with glutamate (5 mM) resulted in an approximate 50% increase in LDH release from the cells, this was significantly reduced by the addition of 10 μg·mL^−1^ *R. nasutus* leaf ethanol extract (ANOVA *post hoc p* value < 0.05). The EC_50_ for prevention HT-22 cells releasing LDH in response to 5 mM glutamate was 5.17 μg·mL ^−1^ (95% CI 1.1–23.3 μg·mL^−1^) *R. nasutus* leaf ethanol extract.

**Figure 7:**
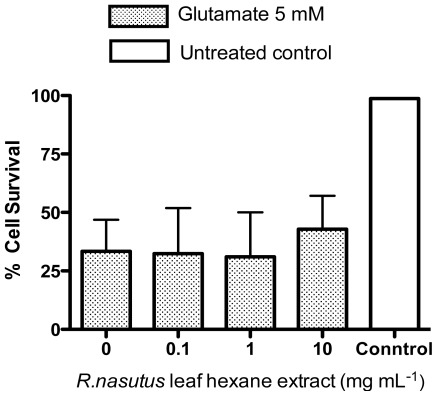
Hexane extract of *R. nasutus* provides no protection against 5 mM glutamate toxicity (Trypan blue exclusion).

The hexane extract of *R. nasutus* leaf was also tested against glutamate toxicity in the HT-22 cells and it was found that this extract provided no protection (between 10 and 0.1 μg·mL^−1^) against 5 mM glutamate (Figure 7).

**Figure 8:**
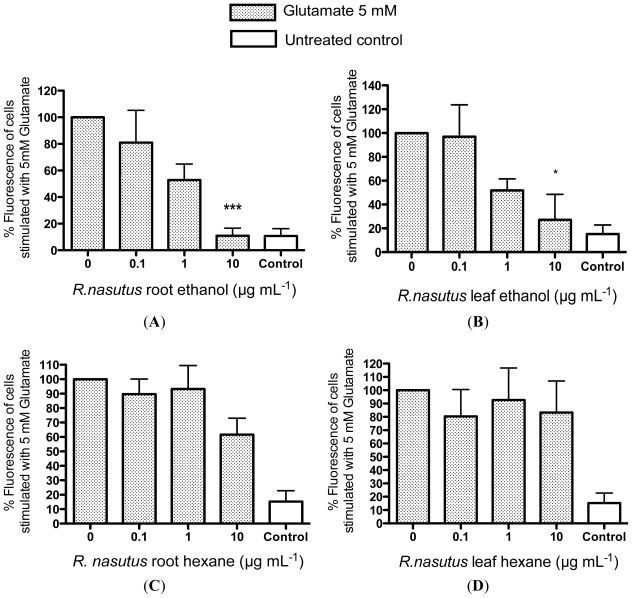
The effect of *R. nasutus* extracts on the production of reactive oxygen species (ROS) after exposure to 5 mM Glutamate. (**A**) The effect of the ethanol extract of *R. nasutus* root on the ROS production in response to 5 mM Glutamate; (**B**) The effect of the ethanol extract of *R. nasutus* leaf on the ROS production in response to 5 mM Glutamate; (**C**) The effect of the hexane extract of *R. nasutus* root on the ROS production in response to 5 mM Glutamate; (**D**) The effect of the hexane extract of *R. nasutus* leaf on the ROS production in response to 5 mM Glutamate. * ANOVA *p* < 0.05; *** ANOVA *p* < 0.001.

Since the mechanism by which glutamate is thought to cause cell death in HT-22 cells (which lack glutamate receptors) is mainly through an increase in oxidative stress, caused by a decrease in glutathione levels, it would make sense that the two ethanol extracts (of leaf and root) from *R. nasutus* appear to be the most effective at preventing toxicity from glutamate, since they had the most antioxidant capabilities (Table 1). In order to confirm that the antioxidant effects seen in Table 1 are translated to cell protection through a reduction of ROS inside the cells we used the intracellular fluorescent ROS dye carboxy-H_2_DCFDA, to measure the increase in ROS in the HT-22 cells and to find out whether the *R. nasutus* extracts were capable of preventing or reducing ROS build up in the cells, preventing the toxicity of the glutamate (Figure 8).

Both the ethanol extracts (root and leaf) of *R. nasutus* (10 μg·mL^−1^) were effective at reducing or preventing the ROS build up in the HT-22 cells in response to glutamate (5 mM), with EC_50_ values of 6.48 μg·mL^−1^ and 1.2 μg·mL^−1^ respectively. However, the hexane extracts (root and leaf) appeared to be ineffective.

Oxidative stress has been implicated in neuron apoptosis found in a number of neurodegenerative disorders. Superoxide radicals, which are produced in the mitochondria during respiration are involved in the generation of many damaging ROS including hydrogen peroxide and hydroxyl radicals [19,26–28]. The data has shown that the extracts, which were most effective at scavenging free radicals such as superoxide, and hydroxyl radicals, (Table 1) were the most effective at reducing the toxicity of 5 mM glutamate by reducing the ROS build up in the cells (Figures 1–7).

**Figure 9:**
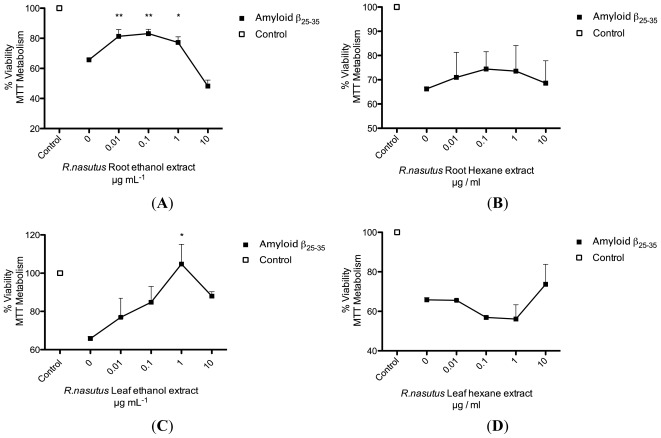
Protective effect of *R. nasutus* extracts against 2 μM amyloid-β_25–35_ toxicity. (**A**) Ethanol extract of *R. nasutus* root; (**B**) Hexane extract of *R. nasutus* root; (**C**) Ethanol extract of *R. nasutus* leaf; (**D**) Hexane extract of *R. nausuts*leaf. * ANOVA Dunnett’s *post hoc* test *p* < 0.05; ** ANOVA Dunnett’s *post hoc* test *p* < 0.01.

Amyloid-β protein is a major constituent of the senile plaques in the brains of Alzheimer’s disease patients, which contribute to the degeneration of the brain via an oxidative stress mechanism, thought to involve and accumulate superoxide and the dismutation of superoxide to hydrogen peroxide [25]. The amyloid-β_25–35_ protein fragment used in our experiments is thought to cause cell death through a necrotic mechanism [29,30]. We have shown that *R. nasutus* is capable of scavenging free radicals (Table 1) and can protect against glutamate toxicity (Figures 1–7) and hypoxia induced cell death [3]. We followed this up by treating the HT-22 cells with 2 μM amyloid-β_25–35_, which consistently resulted in a 30–40% decrease in cell viability (Figure 9). After pretreatment (24 h) with *R. nasutus* ethanol extracts of root or leaf (Figure 9A,C) there was a significant increase in HT-22 cell viability (measured with MTT assay) with the peak being at 0.1 μg·mL^−1^ for the root (ANOVA *p* value<0.01) and 1 μg·mL^−1^ for the leaf (ANOVA *p* value <0.05). The hexane extracts (Figure 9B,D), however, did not show any significant increase in HT-22 cell viability within the concentrations of extract tested.

**Figure 10:**
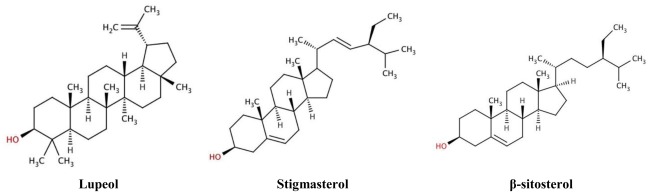
Structures of compounds found in *R. nasutus*, which were quantified using HPLC.

Both the ethanol extracts of *R. nasutus* root and leaf are able to protect the HT-22 cells against both glutamate and amyloid-β toxicity. The hexane extract from the root is able to protect the cells against glutamate but not amyloid-β toxicity. Both the ethanol extracts (from root and leaf) have much higher antioxidant scavenging properties than the hexane extracts (of leaf and root) and this appears to correlate with the flavonoid contents (Table 2). However, despite this the hexane extract of the root is still effective at protecting the HT-22 cells against the glutamate toxicity, even though it has low ROS scavenging activity and a low flavonoid content. It is therefore possible that it provides its protection via a mechanism other than ROS scavenging. Previous studies have investigated the effects of quercetin (a flavonoid found in foods) on amyloid-β toxicity [31–33] and shown that scavenging of ROS can be protective against β-amyloid induced cell death. Other studies have shown that hormones such as estrogen are able to protect neuron cells against glutamate and amyloid-β insults [34]. Estrogen is thought to protect cells in various ways including the activation of growth factors, maintaining plasticity of neurons, and preventing apoptotic pathways, as well as potentially being a free radical scavenger [17,26,35]. Amongst others, the triterpenoid, lupeol and sterols, stigmasterol and β-sitosterol have been previously isolated from *R. nasutus* root [36] (structures found in Figure 10) and could potentially be pharmacologically active in preventing the cell death caused by glutamate or amyloid-β_25–35_ protein. Both β-sitosterol and stigmasterol have the 3-OH group (highlighted in red in Figure 10) thought to be necessary for neuroprotection [17].

**Figure 11:**
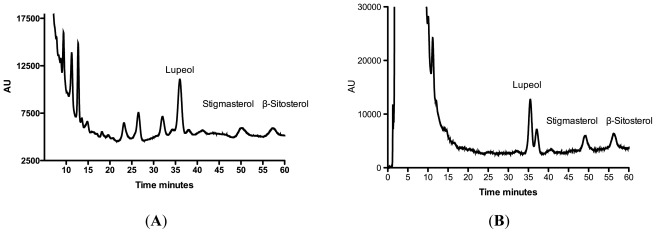

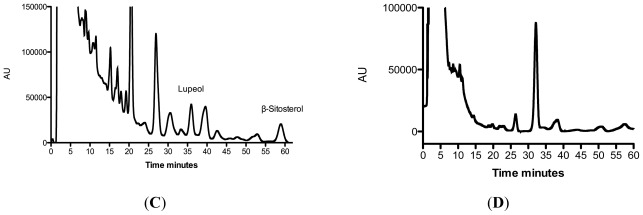
Chromatographs of *R. nasutus* extracts. (**A**) Ethanol extract of *R. nasutus* root (50 mg·mL^−1^); (**B**) Hexane extract of *R. nasutus* root (50 mg·mL^−1^); (**C**) Ethanol extract of *R. nasutus* leaf (50 mg·mL^−1^); (**D**) Hexane extract of *R. nasutus* leaf (50 mg·mL^−1^). Peaks: 1. Lupeol, 2. Stigmasterol, 3. β-sitosterol.

We used high-performance liquid chromatography (HPLC) to quantify the compounds selected in the *R. nasutus* extracts; the average retention times from six separate injections are 35.66 ± 0.16, 50.07 ± 0.09 and 57.53 ± 0.23 for lupeol stigma sterol and β-sitosterol respectively. The chromatograms (representative from at least three injections) for each of the extracts are shown in Figure 11. Using the area under the curve from a range of concentrations of the standards we quantified each of the compounds in the extracts Table 3.

The ethanol extract of *R. nasutus* leaf contained the highest concentrations of lupeol and β-sitosterol. Whereas, the ethanol extract of *R. nasutus* root contained the highest concentration of stigmasterol. The hexane extract of *R. nasutus* root, contained a lower concentration of all of the three tested compounds and the hexane extract of *R. nasutus* leaf did not contain any of the compounds at a concentration that could be detected with HPLC. The lupeol, stigmasterol or β-sitosterol, content appeared to follow the pattern of protection against glutamate and amyloid-β toxicity. The ethanol extracts from leaf and root, which were the most effective at protecting HT-22 cells, contained the highest concentrations of the compounds tested. In the hexane extract of *R. nasutus* leaf there were no detectable levels of lupeol, stigmasterol or β-sitosterol, as well as low ROS scavenging capabilities. The hexane extract of the leaf afforded no protection for the HT-22 cells against glutamate or amyloid-β toxicity. In contrast the hexane extract of the root, which had low ROS scavenging activity and phenolic content, contained a detectable amount of each of lupeol, stigmasterol and β-sitosterol, and was able to provide protection against glutamate toxicity, although not significantly against amyloid-β induced cell death. It is possible that lupeol, stigmasterol, and β-sitosterol could provide some of the protection seen from the *R. nasutus* extracts. Further research into these compounds, as well as others found in *R. nasutus* is ongoing.

**Figure 12:**
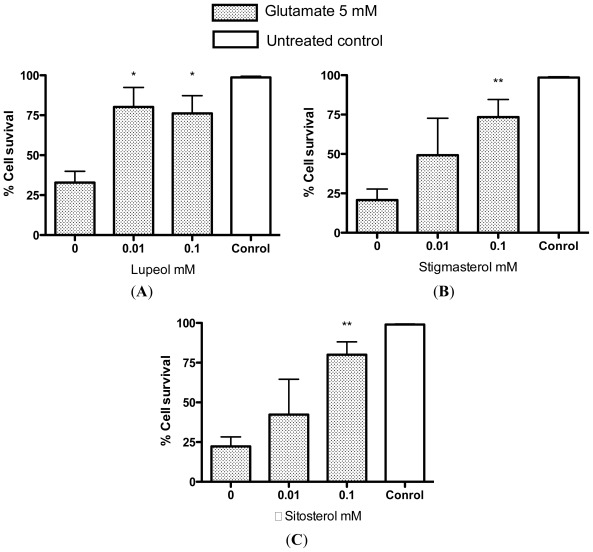
Protection of HT-22 cells against glutamate toxicity by (**A**) lupeol, (**B**) stigmasterol, (**C**) β-sitosterol. Measured using trypan blue exclusion assay. * ANOVA Dunnett’s *post hoc* test *p* < 0.05; ** ANOVA Dunnett’s *post hoc* test *p* < 0.01.

In order to quickly assess the potential protective effects of these compounds, which have been identified as constituents of *R. nasutus* root and leaf extracts, we tested them in the trypan blue exclusion assay. HT-22 cells were co-treated with glutamate (5 mM) and varying concentrations of lupeol, stigmasterol or β-sitosterol. The protective effect was measured 24 h later using trypan blue exclusion as the criteria for cell survival (Figure 12).

**Figure 13:**
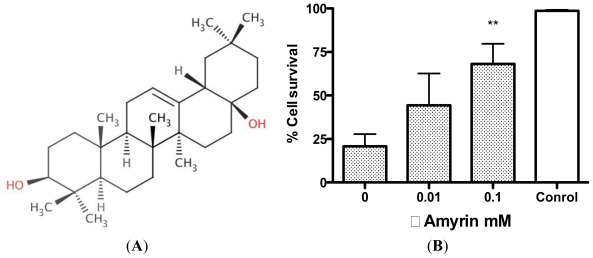
(**A**) β-Amyrin structure (**B**) β-amyrin protection against glutamate toxicity in HT-22 cells. ** ANOVA Dunnett’s *post hoc* test *p* < 0.01.

All three of the tested compounds provided some protection against 5 mM glutamate, with stigmasterol and β-sitosterol showing significant protection at 100 μM, and lupeol providing significant protection at 10 μM. Whilst these protective effects provided by lupeol, stigmasterol and β-sitosterol are interesting, it seems unlikely that any of them are individually responsible for the protection of the HT-22 cells against glutamate or amyloid-β provided by *R. nasutus*, since the concentrations required to give significant protection against glutamate toxicity appear higher than those identified in the extracts with HPLC. However, there could be a cumulative effect of these and other active compounds found in the extract. Another molecule reported to be found in *R. nasutus* extracts is β-amyrin [37]. β-amyrin has previously been shown to be biologically active in other herbal extracts, acting as an antidepressant [20,21]. We therefore tested β-amyrin to see if it could also provide neuroprotection against glutamate toxicity (Figure 13).

The β-amyrin treatment provided significant protection against 5 mM glutamate toxicity at 100 μM. Previous studies have suggested that β-amyrin interacts with the GABA-A receptor [38–40], and causes the release of noradrenalin from newly synthesized pools [20], which in turn has been shown to be protective against glutamate and amyloid-β toxicity in primary cultured rat cortical neurons [41]. It is possible that lupeol, which has been reported to have a number of biological activities including hetatoprotective and tumor-preventative properties [42], stigmasterol and β-sitosterol could have similar actions, contributing to the neuroprotection provided by *R. nasutus*. It remains to be seen if *R. nasutus* extracts or lupeol, stigmasterol and β-sitosterol are able to act as antidepressants, but it is possible they have potential as neuroprotective agents in neurodegenerative diseases such as Alzheimer’s diseases, which have oxidative stress as a component of the neuron loss.

## 3. Experimental Section

### 3.1. Chemicals

Stigmasterol, β-amyrin, lupeol and β-sitosterol were purchased from Sigma-Aldrich (St. Louis, MO, USA). The HPLC grade methanol was obtained from Burdick & Jackson (Muskegon, MI, USA).

### 3.2. Amyloid-β_25–35_

Amyloid-β_25–35_ 2 μM (Sigma-Aldrich, St. Louis, MO, USA) was dissolved in sterile phosphate buffered saline (PBS) and stocks frozen at −20 °C. New amyloid-β_25–35_ and aged amyloid-β_25–35_ have both been shown to readily form fibrils and cause equal toxicity to cultured cells [43]. The stocks were diluted in serum free media and incubated at 37 °C for at least 1 h before addition to cells.

### 3.3. Cell Culture

HT-22 cells, a kind gift from Professor David Schubert at the Salk Institute (San Diego, CA, USA), were maintained in Dulbecco’s Modified Eagle Medium (DMEM) (Invitrogen, Carlsbad, CA, USA) supplemented with 10% fetal bovine serum (FBS) and antibiotics (penicillin and streptomycin) and were incubated at 37 °C in a humidified 5% CO_2_ atmosphere.

### 3.4. Plant Material

*Rhinacanthus nasutus* (L.) Kurz was collected from the Princess Maha Chakri Sirindhorn Herbal Garden (Rayong Province, Thailand) in March 2010, and authenticated by Thaweesakdi Boonkerd (Department of Botany, Faculty of Science, Chulalongkorn University, Thailand). The voucher specimen [013416 (BCU)] was deposited at Kasin Suvatabhandhu Herbarium, Department of Botany, Faculty of Science, Chulalongkorn University, Thailand. *R. nasutus* was dried and ground into a fine powder before extraction in ethanol or hexane. The supernatant was subsequently filtered and the ethanol/hexane evaporated using a rotating evaporator at (60 °C). The resulting product was suspended in dimethyl sulfoxide (DMSO) at a concentration of 100 mg·mL^−1^, this could then be mixed in aqueous solutions at the concentrations required for the experiments.

### 3.5. Trypan Blue Exclusion Assay

HT-22 cells were plated in six well culture plates at a density of 1 × 10^4^ cells per well and allowed to adhere overnight, in a volume of 2 mL of DMEM containing 10% FBS. The following day cells were treated with neurotoxin (glutamate 5 mM), *R. nasutus* extract (0.1 mg·mL^−1^, 1 mg·mL^−1^ or 10 mg·mL^−1^), or a combination of the two. The cells were then returned to the 37 °C in a humidified 5% CO_2_ incubator for 18 h. The media was then removed, and the cells stained with 0.2% trypan blue (Gibco, Invitrogen, Carlsbad, CA, USA) in PBS for 3 min. The excess trypan blue was removed and replaced with PBS to prevent the cells from drying out. The cells were then viewed under 20 times magnification objective, and the percentage of living cells counted by a 2nd individual who had no prior knowledge of the treatments, over five randomly selected, non-over lapping fields of view. Trypan blue is not eliminated by dead cells, therefore the blue cells were counted as dead and non-blue cells as alive.

### 3.6. 3-(4,5-Ethylthiazol-2-yl)-2,5-diphenyltetrazolium Bromide (MTT) Assay

HT-22 cells were plated into 96 well culture plates at a density of 3000 per well, in 100 μL DMEM supplemented with 10% FBS. The cells were allowed to adhere over night in the 37 °C humidified 5% CO_2_ incubator. The following day the cells were treated as stated, with neurotoxin (amyloid-β_25–35_ 2 μM (Sigma-Aldrich, St. Louis, MO, USA), or glutamate 5 mM) herb or a combination of the two, as well as untreated controls, and wells treated with 100% DMSO to provide a reading for 100% cell death, before the cells were returned to the incubator for 18 h. MTT reagent was purchased from Merck (Hohenbrunn, Germany) and dissolved in sterile PBS at a stock concentration of 12 mM (5 mg mL^−1^), 20 μL of the stock MTT was added to the well resulting in a final concentration of 1 mM (0.45 mg mL^−1^) MTT in each well. The plate was then returned to the incubator at 37 °C in a humidified 5% CO_2_ atmosphere for four hours. After this incubation period the media was carefully removed, and the formazan crystals solubilized in 100 μL of DMSO and mixed by pipetting up and down. The absorbance of each sample was measured at 550 nm using a microplate reader. MTT reduction measured at 550 nm was converted to percentage cell growth was determined using the following formula.

%Cell MTT metabolism=(Sample Abs 550 nm-Blank Abs 550 nm)/Control Abs 550 nm

### 3.7. Lactate Dehydrogenase Release (LDH) Assay

HT-22 cells were plated at a density of 10^4^ cells per well in 96 well plates and allowed to adhere overnight in a humidified 37 °C, 5% CO_2_ atmosphere. The following day the media was changed and the cells treated with neurotoxin (glutamate 5 mM) with or without *R. nasutus* extract, before being incubated at 37 °C in a humidified 5% CO_2_ atmosphere for a further 18 h. To assay the LDH release form the HT-22 cells the CytoTox 96 Non-Radioactice Cytotoxixity Assay (Promega) was used as per the manufactures instructions. Fifty μL of media was transferred to a fresh microtitre plate from the treated cells, as well as a set of control and lysed cells (to determine the background, and maximum release of LDH). Fifty microliters of assay substrate solution was added to this, and the plate was then incubated in the dark for 30 min. The stop solution was added (50 μL) and the plate absorbance read at 590 nm in a micro plate reader.

### 3.8. Total Phenolic Content Assay

Total phenolic content in *R. nasutus* extracts was measured using the Folin-Ciocalteu method [40] modified for a 96 well plate reader, using gallic acid to generate a standard curve (100–1.56 μg·mL^−1^). Folin-Ciocalteu reagent was diluted; one in ten, in deionized water and 50 μL was incubated with the herb (50 μL) at various concentrations between (1 and 0.25 mg·mL^−1^) for 20 min in the dark. The solutions were then neutralized with 35 μL Na_2_CO_3_ before a further 20 min incubation period, followed by reading in a micro plate reader (abs 750 nm). The total phenolic content is represented as gallic acid equivalents (GAE) mg g^−1^ of dry extract.

$$\begin{array}{c} \text{Abs }750=0.013\times {\text{C}}_{\text{Gallic acid}}\;(\mu \text{g }{\text{mL}}^{-1})+0.0129\\ R^{2}=0.997 \end{array}$$

### 3.9. Total Flavonoid Content Assay

The total flavonoid content was determined using an aluminum chloride colorimetric method [40] modified for a 96 well plate reader, using rutin hydrate as to generate a standard curve (100 μg·mL^−1^ to 0.7 μg·mL^−1^). Per well, 5 μL of 10% aluminum chloride hexahydrate, 5 μL 1 M potassium acetate and 140 μL deionized water were incubated with 50 μL herb (0.5 to 5 mg·mL^−1^) or rutin and incubated for 40 min in the dark. The absorbance at 415 was measured in a micro plate reader. The total flavonoid content is represented as rutin equivalents (RE) mg·g^−1^ of dry extract.

$$\begin{array}{c} \text{Abs }415=0.004\times {\text{C}}_{\text{Rutin}}\;(\mu {\text{g}\cdot \text{mL}}^{-1})+0.004\\ R^{2}=0.997 \end{array}$$

### 3.10. 2,2′-Azino-*bis*(3-ethyl)benzothiazoline-6-sulphonic Acid (ABTS) Decolorization Assay

The ABTS assay is based on the absorbance of the ABTS^+^ radical at 734 nm, which is a blue color, however, in the presence of antioxidants the ABTS^+^ is returned to the colorless ABTS. The green/blue stable ABTS^+^ radical is formed by mixing ABTS (2 mM) with potassium persulphate (17 mM) (50:0.3 mL) and allowing the mixture to stand at room temperature in the dark overnight [40]. On the day of the assay the ABTS^+^ solution was diluted such that it gave an absorbance of between 0.7 and 0.8 at 734 nM. Herb extracts at a range of concentrations (10–0.1 μg·mL^−1^) were incubated with ABTS^+^ in 96 well plates (final volume 200 μL) for 30 min before the absorbance was measured at 734 nm. l-Ascorbic acid was used as a standard antioxidant (2–0.2 μg·mL^−1^) in the same way as the herb extracts.

### 3.11. Hydroxyl Radical Scavenging Assay

Hydroxyl radicals were generated by a Fenton reaction system, and the scavenging capacity towards the hydroxyl radicals was measured by using a deoxyribose method [41]. The reaction mixture contained, 50 μL of sample (in phosphate buffered saline pH = 7.4), 8 μL of EDTA (1.04 mM), 8 μL of FeCl_3_ (1.0 mM) and 8 μL of 2-deoxyribose (60 mM). The mixtures were incubated at 37 °C and the reactions were initiated by adding 8 μL of ascorbic acid (4 mM) and 8 μL of H_2_O_2_ (10 mM). After incubation at 37 °C for 1 h, 80 μL of thiobarbituric acid (10 g·L^−1^) were added into the reaction mixture followed by 80 μL of HCl (25%). The mixtures were then heated to 95 °C for 15 min and then allowed to cool. The absorbance of the solution was measured at 532 nm with a spectrophotometer. Hydroxyl radical scavenging capacity was evaluated with the percentage inhibition of 2-deoxyribose oxidation by hydroxyl radicals. The scavenging percentage was calculated according to the following formula:

$$\%\text{hydroxyl radical scavenged}=\frac{\left[100\times (A-B)-C\right]}{C}$$

where: *A* = sample absorbance 532 nm; *B* = blank absorbance 532 nm; *C* = control absorbance 532 nm.

### 3.12. Nitric Oxide Scavenging Assay

Nitric oxide was generated from sodium nitroprusside and was measured using the Griess reagent. An aqueous solution of sodium nitroprusside at pH 7.4 spontaniously generates nitric oxide, which reacts with oxygen to form nitrite ions [42]. *R. nasutus* extracts diluted in PBS (1–0.125 mg·mL ^−1^) were incubated with sodium nitropursside 10 mM, for 3 h, in the dark at room temperature. Nitric oxide production was then measured using the Griess reagent, (2% sulphanilamide, 0.1% naphthylethylenediamine dichloride and 3% phosphoric acid). The color change of the reaction was measured at 546 nm. Ascorbic acid was used as a positive control.

### 3.13. Superoxide Scavenging Assay

Superoxide radicals are precursors to a number of ROS including H_2_O_2_. Superoxide free radicals were formed using alkaline DMSO (1 mL of air saturated DMSO containing 5 mM NaOH in 0.1 mL H_2_O), which reacts with Nitro-blue tetrazolium (NBT) to produce red colored diformazan salt at room temperature. Superoxide scavengers are capable of inhibiting the formation of the red formazan product [44]. The herb extracts (diluted in PBS 10–0.1 μg·mL^−1^) were incubated with the alkaline DMSO NBT mixture for 30 min at room temperature in the dark, in a volume of 200 μL in a microtitre plate, the absorbance was then read at 560 nm.

### 3.14. 2,2-diphenyl-1-Picrylhydrazyl (DPPH) Assay

2,2-diphenyl-1-picrylhydrazyl (DPPH) was dissolved in methanol at a concentration of 200 μg·mL^−1^, this was added to a microtitre plate at a 1:1 ratio with the *R. nasutus* sample with a final volume of 100 μL. The *R. nasutus* extracts were diluted in methanol and blanks were prepared for the herb extracts at 1:1 ratio with methanol replacing the DPPH solution. The microtitre plate also contained a 100% DPPH (DPPH solution 1:1 methanol). The plate was incubated in the dark for 30 min before the absorbance was measured at 517 nm in a microplate reader. Percentage DPPH radical scavenging was calculated using the equation below.

$$\%\text{DPPH Scavenged}=\frac{\left[100\times (A-B)-C\right]}{C}$$

where: *A* = Sample absorbance 517 nm; *B* = Blank absorbance 517 nm; *C* = DPPH control absorbance 517 nm.

### 3.15. Carboxy-H_2_-DFCDA Free Radical Assay

HT-22 cells were cultured in 96 well plates in 100 μL of DMEM overnight at 37 °C in a humidified 5% CO_2_ atmosphere. The following day the media was changed and drugs added, glutamate at 5 mM, and *R. nasutus* at 0.1, 1 and 10 μg·mL^−1^ before being returned to the 37 °C in a humidified 5% CO_2_ atmosphere over night. The following day the media was removed and the cells washed with warm HBSS, before loading with 10 μM Carboxy-H_2_-DFCDA at 37 °C for 45 min. The cells were then washed 3 times with warm HBSS and the fluorescence measured at emission 521 nm after excitation at 494 nM.

### 3.16. High Performance Liquid Chromatography

HPLC was conducted with a Shimadzu chromatograph, equipped with 2 LC-10AT Shimadzu pumps, an SPD-10Ai UV-Vis detector and a Pinnacle II C18, 5 μm, 10 × 4.6 mm column housed in a Shimadzu CTO-10A oven set to 30 °C. The mobile phase was 92.5% methanol 7.5% water, with a flow rate of 1 mL·min^−1^. Samples were loaded manually in a volume of 20 μL; data were recorded at wavelength 210 nm with LC Solution software (version 1.25; Shimadzu Corporation: Kyoto, Japan, 2002–2009).

### 3.17. Data Analysis

Data was analyzed using Graphpad Prism Software (version 5 for Mac; Graphpad software Inc.: San Diego, CA, USA, 2010). Means from three or more independent experiments are expressed plus or minus the standard error (or with 95% confidence intervals for EC_50_ data presented on a log scale), and statistical analysis of significance was tested with ANOVA followed by the Dunnett’s *post hoc* test.

## 4. Conclusions

The *R. nasutus* extracts from root and leaf contain varying amounts of phenolic and flavonoid compounds, as well as varying concentrations of the triterpenoidlupeol, and the sterols stigmasterol and β-sitosterol. The ethanol extracts of the leaf and root have high free radical scavenging capabilities, as well as protecting HT-22 cells against glutamate and amyloid-β toxicity. This protection is likely to be as a result of a combination of different types of compounds, possibly acting though more than one mechanism, possibly including free radical scavenging, caspase inhibition or growth factor production. The hexane extract of *R. nasutus* root had low free radical scavenging activity and a low phenolic and flavonoid content, yet it was still able to protect HT-22 cells against glutamate toxicity although the protection in the amyloid-β toxicity assay was not significant. The hexane extract of *R. naustus* root does have a moderate amount of each of the compounds assayed using HPLC so it might possible that these compounds (lupeol, stigmasterol and β-sitosterol) play a larger role in the protection of HT-22 cells against the glutamate, although it is not yet clear whether they can protect against amyloid-β toxicity.

In conclusion we have identified *R. nasutus* as a neuroprotective agent, which contains a number of potentially pharmacologically active compounds, further research into these and other compounds is required to discover the full extent of their potential uses as neuroprotective agents.

## Figures and Tables

**Table 1:** Radical scavenging capabilities of *R*hinacanthus *nasutus* extracts. The data is represented as the PEC_50_ (g·mL^−1^), which is the −Log_10_ of the dose required for 50% maximal scavenging of the radical.

*R. nasutus* Extract	Hydroxyl Scavenging Assay	*n*	ABTS^+^ Decolorization Assay	*n*	DPPH Scavenging Assay	*N*	NO^−^ Scavenging Assay	*n*	SO Scavenging Assay	*n*
Root Ethanol	4.5 ± 0.38	3	3.49 ± 0.07	3	3.5 ± 0.15	4	<2	2	3.25 ± 0.22	3
Root Hexane	ND	3	3.28 ± 0.43	3	1.9 ± 0.01	5	<2	2	2.81 ± 0.03	3
Leaf Ethanol	4.4 ± 0.27	3	4.04 ± 0.06	3	4.4 ± 0.05	4	<2	2	4.49 ± 0.07	3
Leaf Hexane	ND	3	2.30 ± 2.40	3	0.5 ± 0.10	3	<2	2	2.31 ± 0.20	3
Trolox	3.2 ± 0.08	3	3.60 ± 0.02	3	4.9 ± 0.02	5	NA	0	NA	0
L-Ascorbic Acid	4.3 ± 0.15	3	3.86 ± 0.10	3	6.1 ± 0.11	3	±	2	6.14 ± 0.05	3

ND = Not detected within the range of the assay; NA = Not assayed.

**Table 2:** Total phenolic and flavonoid content of *R. nasutus* extracts.

*R. nasutus* Extract	Total Phenolic Content GAE mg·g^−1^ Dry Extract	*n*	Total Flavonoid Content RE mg·g^−1^ Dry Extract	*n*
Root Ethanol	2.17 ± 0.15	5	1.13 ± 0.17	4
Root Hexane	0.36 ± 0.11	5	ND	4
Leaf Ethanol	6.50 ± 0.12	5	1.15 ± 0.14	4
Leaf Hexane	0.40 ± 0.01	5	ND	4

ND = Not detected within the range of the assay.

**Table 3:** Concentration of compounds in *R. nasutus* extracts from standard curves using HPLC.

*R. nasutus* Extract	Lupeol μg·mg^−1^ Extract	Stigmasterol μg·mg^−1^ Extract	β-sitosterol μg·mg^−1^ Extract
Root ethanol	5.4 ± 0.3	13.7 ± 0.1	0.5 ± 0.1
Root hexane	1.5 ± 0.3	2.91 ± 0.03	1.3 ± 0.2
Leaf ethanol	12.4 ± 2.5	ND	17.4 ± 3.2
Leaf hexane	ND	ND	ND

ND = Not detected.
